# Comparative genome analysis using sample-specific string detection in accurate long reads

**DOI:** 10.1093/bioadv/vbab005

**Published:** 2021-05-31

**Authors:** Parsoa Khorsand, Luca Denti, Human Genome Structural Variant Consortium, Paola Bonizzoni, Rayan Chikhi, Fereydoun Hormozdiari

**Affiliations:** 1 Genome Center, UC Davis, Davis, CA 95616, USA; 2 Department of Computational Biology, Institut Pasteur, Paris 75015, France; 3 Department of Informatics, Systems and Communication, University of Milano-Bicocca, Milano, 20126, Italy; 4 UC Davis MIND Institute, Sacramento, CA 95817, USA; 5 Department of Biochemistry and Molecular Medicine, Sacramento, UC Davis, Sacramento, CA 95817, USA

## Abstract

**Motivation:**

Comparative genome analysis of two or more whole-genome sequenced (WGS) samples is at the core of most applications in genomics. These include the discovery of genomic differences segregating in populations, case-control analysis in common diseases and diagnosing rare disorders. With the current progress of accurate long-read sequencing technologies (e.g. circular consensus sequencing from PacBio sequencers), we can dive into studying repeat regions of the genome (e.g. segmental duplications) and hard-to-detect variants (e.g. complex structural variants).

**Results:**

We propose a novel framework for comparative genome analysis through the discovery of strings that are specific to one genome (‘samples-specific’ strings). We have developed a novel, accurate and efficient computational method for the discovery of sample-specific strings between two groups of WGS samples. The proposed approach will give us the ability to perform comparative genome analysis without the need to map the reads and is not hindered by shortcomings of the reference genome and mapping algorithms. We show that the proposed approach is capable of accurately finding sample-specific strings representing nearly all variation (>98%) reported across pairs or trios of WGS samples using accurate long reads (e.g. PacBio HiFi data).

**Availability and implementation:**

Data, code and instructions for reproducing the results presented in this manuscript are publicly available at https://github.com/Parsoa/PingPong.

**Supplementary information:**

Supplementary data are available at *Bioinformatics Advances* online.

## 1 Introduction

Whole-genome sequencing (WGS) has become the dominant approach in studying variations across genomes. Today, WGS data continue to provide invaluable insight into every aspect of biology. In particular, comparative analysis of multiple samples using WGS data is fundamental in understanding the genetics of disorders, traits and evolution. The comparison of differences found between exome and genome of affected cases and unaffected controls has successfully found genetic variants associated with disorders and guided predicting genes contributing to disorders (Cirulli and Goldstein, 2010). Population genomics studies benefit from WGS data by finding shared or discriminative sequences and genomic variants between different populations (1000 Genomes Project Consortium *et al.*, 2015; Mallick *et al.*, 2016). Furthermore, evolutionary studies also benefit from such comparative studies in a multiple species setting (Prado-Martinez *et al.*, 2013; Genome 10K Community of Scientists, 2009).

High-throughput short-read sequencing (i.e. Illumina) has been the driving force behind most of the WGS studies in the past decade. Short-read sequencing is cheap, provides high-throughput data and has a low error rate (Shendure and Ji, 2008). However, it also has several major drawbacks. First, the assembly of the eukaryotic genomes using short-read sequencing data is nontrivial and computationally resource-intensive (Kingsford *et al.*, 2010). Second, the short length of the reads (generally below 250 bp) produced by these technologies has caused significant complexity and ambiguity in studying repeat regions of the genome (Chaisson *et al.*, 2015; Logsdon *et al.*, 2020; Zook *et al.*, 2020). Third, the quality of structural variation (SV) and other complex variant calls predicted using short-reads data has remained low despite significant bioinformatics efforts and still requires orthogonal validations (Chaisson *et al.*, 2019; Soylev *et al.*, 2019). Finally, several types of genetic variations are hard to predict using short-read sequencing technologies due to their repeat nature (e.g. VNTR expansions; Bakhtiari *et al.*, 2020).

With the advent of long-read sequencing technologies (e.g. PacBio or Oxford Nanopore), we have access to long reads (>10 kb) that can be used to overcome the above-mentioned shortcomings of short-read sequencing (Bzikadze and Pevzner, 2020; Chaisson *et al.*, 2019; Miga *et al.*, 2020). WGS data from long-read sequencing technologies enable one to discover and further study variants that were either hidden or unreliably predicted from short-read data. For example, a recent benchmark showed that long-read sequencing data enabled to find a large fraction (over 50% of SVs), which were unreported from short-read sequencing data (Chaisson *et al.*, 2019, 2015; Zook *et al.*, 2020).

One of the main objectives of performing WGS is the comparison of two or more genomes. Such comparative genomics studies are concerned with multiple individuals from the same species or from multiple species, either in a case versus control setting or within population genomics (Karlin *et al.*, 1998). The discovery of differences between multiple samples using WGS is at the core of most genomic analysis.

The dominant framework for comparative variant analysis among multiple sequenced samples is based on mapping the reads to the reference genome (Langmead and Salzberg, 2012; Li, 2018), predicting variants in each sample and comparing the predicted loci (Albers *et al.*, 2011; Chaisson *et al.*, 2015; Medvedev *et al.*, 2009; Poplin *et al.*, 2017). The comparison of variants predicted between multiple samples is based on overlapping the predicted variant locations. This strategy is effective for comparing SNVs; however, for many SVs the exact breakpoint position is hard to establish and ambiguities can negatively affect accuracy. There are several heuristics used for comparing SVs in multiple samples by considering that the exact breakpoint for the SV might not be known or ambiguous (https://simpsonlab.github.io/2015/06/15/merging-sv-calls/). These are based on merging SVs with approximately close breakpoints and considering reciprocal overlaps as a match (Chaisson *et al.*, 2019). Such heuristics tend to work for SVs in simple regions of the genome. However, for more complex scenarios such as STR/VNTR expansions (Bakhtiari *et al.*, 2018; Gymrek *et al.*, 2012), SVs with adjacent single nucleotide polymorphism (SNP) variants (Cameron *et al.*, 2019) or SVs with breakpoints in repeats (e.g. segmental duplications) will result in reduction of accuracy as these heuristics tend to fail (Chaisson *et al.*, 2019; Numanagić *et al.*, 2018; Soylev *et al.*, 2019).

An alternative approach for comparative genome analysis is not to directly compare the predicted variants among multiple samples but rather to find the *sequences containing breakpoints* that are different between samples. This approach can be implemented without the need to map the reads to the reference genome and predict variants per sample (i.e. mapping-free approaches). Examples of mapping-free approaches for studying genomes are DiscoSNP++ (Peterlongo *et al.*, 2017), Scalpel (Narzisi *et al.*, 2014), VarGeno (Sun and Medvedev, 2019), MALVA (Denti *et al.*, 2019), Nebula (Khorsand and Hormozdiari, 2021) and HAWK (Rahman *et al.*, 2018) for detection and genotyping of variants in WGS data, and DE-Kupl (Audoux *et al.*, 2017) for detecting RNA-Seq variations. These mapping-free approaches have the advantage of not being impacted by the possibility of ambiguity in SV breakpoints or inaccuracies in the reference genome itself. The mapping-free approaches developed for studying short-read sequencing data are mostly based on finding *k*-mers that distinguish one sample from other samples. The idea of computing *k*-mers that are unique to a target w.r.t. a background set of genomes is also proposed in Phillippy *et al.* (2007). In general, the length of *k*-mers (i.e. *k*) is a fixed constant and usually short. However, for long and accurate reads, we are not limited by the length of the short reads and can select arbitrarily long *k*-mers if needed. This flexibility on the length of sequences selected can be advantageous for comparative studying of repeat regions of the genome. The tools mentioned above are fundamentally unable to deal with variable-length *k*-mers and therefore novel developments are needed to fully explore this direction.

We propose a novel method for comparative analysis of multiple WGS samples using accurate long-read sequencing data (e.g. HiFi reads from PacBio; Wenger *et al.*, 2019), without the need to map the reads to a reference genome or choose a fixed *k* value. The advantages of utilizing flexible length strings (e.g. adaptive seeds) in pattern matching have been previously demonstrated (Kiełbasa *et al.*, 2011). The main novelty is the formulation and the resolution of a new computational problem concerned with enumerating sample-specific strings, while avoiding a combinatorial explosion due to the quadratic size of the set of potential candidates. We show that this approach enables identifying nearly all sequences spanning variants between two human genomes on actual PacBio HiFi data. Some of the applications of the proposed comparative genome analysis framework include finding *de novo* variants, sequences segregating in a pedigree or markers distinguishing between populations (e.g. cases and controls).

## 2 Problem definition

Consider two sets of strings: *T* (targets) and *R* (references). Here by *references*, we mean either (i) a reference genome or (ii) a set of unassembled reads that are coming from an unknown reference genome or (iii) a heterogeneous set of reads and genomes that are taken together to be the reference pangenome of some population of interest. We are interested in enumerating substrings of the targets that do not appear as exact substrings of the references.

As a motivating example consider two individuals and their respective sets of sequencing reads *T* and *R*. We define a *variant* as a genomic event that can be described by a single line in the VCF format, such as an SNP, an insertion or deletion or a structural variant such as a duplication or a translocation. More complex forms of genomic variation, e.g. inversion-duplications, can be seen as combinations of variants and therefore are not further considered here. The intuition is that for each variant, there should exist at least one substring of the genome of *T* spanning this variant that is not found within the genome of *R*. Indeed, the whole genome of *T* would be one such substring, but there also likely exist shorter strings than that. Translating this observation to reads, there should exist for each variant at least one substring of *T* that is not found in *R*. We postulate, and will later experimentally verify, that with long and accurate enough reads virtually all variants can be found in substrings of *T* that do not appear in *R*.

We are now returning to the abstract formulation of our initial problem of finding substrings of the targets not found in the references. For two strings *s* and *t*, we will use the notation s⊏t to indicate that *s* is a substring of *t* (and s⊏t for *s* is not a substring of *t*). Formally, we want to enumerate the set & of all strings *s* such that

1. there exists t∈T where s⊏t, and
2. for all r∈R, s⊏r.

In the worst case, the size of & can be quadratic in the total length of strings in *T*, which is too large to be stored or even enumerated. Therefore, we will instead seek a reduced set of strings *S_T_* that can be seen as a minimal representation of & that do not consider strings having proper substrings in & (the *substring-free* property). This is formalized as the following problem:

Problem 1 [Substring-Free Sample-specific (SFS) strings]. Let *T* and *R* be two sets of strings, targets and references, respectively*. Let*&*be the set of all strings satisfying conditions 1 and 2 above. Return the largest subset*$S_{T}\subset \;$*such that for all*$s\in S_{T}$*, there does not exist*$s\prime \in S_{T},s\prime \neq s$*, where*s′⊏s*; i.e.S_T_ is the set of all strings from*&*for which no shorter string of*&*isasubstring of them.*

A string $s\in S_{T}$ is then called a *T-specific string* w.r.t. references *R*, or simply *specific string* when *T* and *R* are clear from the context. Furthermore, a *T*-specific string *s* that is a substring of t∈T, will be also called a *t*-specific string. In the following, we will sometimes omit recalling that *T*-specific (and *t*-specific) strings are substring-free.

**Fig. 1.**
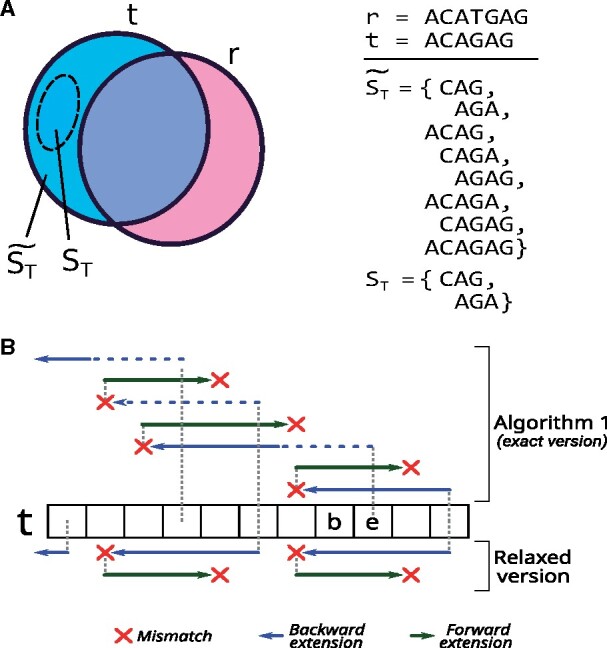
(**a**) Illustration of the SFS framework. Consider a target string *t* and a reference string *r*, each represented by a circle symbolizing all substrings. Blue area: substrings of *t* not in *r*; pink: substrings of *r* not in *t*; purple: substrings common to both *t* and *r*. We start by enumerating &, consisting of all strings *s* that satisfy conditions 1 and 2 of Section 2 (i.e. *s* is a substring of *t* and not a substring of *r*). Then, the set *S_T_* (result of SFS) is the largest substring-free subset of &. (b) The Ping-pong search algorithm (top) starts from the end of the input string *t* and alternates between backward and forward extensions. When the backward extension (blue arrows) ends due to a mismatch (red cross), the algorithm starts a forward extension (green arrows) until another mismatch is found. After a single iteration (outer while loop of the pseudocode), a *t*-specific string t[b−1:e+1] is found and the algorithm restarts the search from position *e*, allowing solutions to ‘overlap’ on *t*. A dashed blue line represents bi-intervals that were already computed during a forward search (and therefore not recomputed in the next iteration). In the relaxed version of the algorithm (bottom side), solutions cannot overlap and the search restarts from position *b—*2 instead of *e*. We note that Algorithm 1 outputs substring t[b:e] since *b* (resp. *e*) has been already decremented (resp. incremented)

We will refer to Problem 1 as the ‘SFS problem’, and an instance is illustrated in Figure 1a. It is easy to see that SFS can be (inefficiently) solved in $O(n^{3})$ worst-case time and $O(m^{3})$ memory, where *n* and *m* are the total lengths of strings in *T* and *R*, respectively. The set & can be constructed by enumerating all substrings of *T* and checking their membership in a hash table containing all substrings in *R*; then another pass over & constructs *S_T_* in linear time and space over the total length of strings in &, e.g. through indexing & using an FM-index. In this paper, we will propose a novel and more efficient quadratic-time $O(n^{2})$ algorithm (Algorithm 1 in Section 4) using linear space *O*(*m*) for solving the SFS problem. We will also propose a heuristic version of the algorithm that solves a relaxed variant of Problem 1 in linear time *O*(*n*). All these complexities are on top of the FMD-index construction (Li, 2012), which in our case can be done in *O*(*m*) time and space (Belazzougui *et al.*, 2020).

The following property shows that it is sufficient to consider instances of the SFS problem where *T* is reduced to a single string.

Property 1 (Local substring-free property). *Let T and R be two sets of strings (targets and references, respectively). The set S_T_ of T-specific strings w.r.t. R, i.e.the solution of SFS problemcan be computed as the union of the sets S_t_ with*t∈T*, where S_t_ is the set of t-specific strings.*

Proof. For the sake of simplicity, assume that $T=\{t_{1},t_{2}\}$. Let $S_{t_{1}}$ be the set of *t*_1_-specific strings obtained as a solution of the SFS problem on instance $\left(\{t_{1}\},R\right)$ and similarly let $S_{t_{2}}$ be the set of *t*_2_-specific strings on instance $\left(\{t_{2}\},R\right)$. We need to prove that given *S_T_* the solution of the SFS problem on instance $\left(\{t_{1},t_{2}\},R\right)$, then $S_{T}=S_{t_{1}}\cup S_{t_{2}}$. Let us first observe that $S_{T}\subseteq S_{t_{1}}\cup S_{t_{2}}$ as indeed each string *s* in *S_T_* must be a substring of *t*_1_ or of *t*_2_ and thus *s* is a *t*_1_-specific string or is a *t*_2_-specific string. Hence let us now prove that $S_{t_{1}}\cup S_{t_{2}}\subseteq S_{T}$. By construction, any *t*_1_-specific string (as well as any *t*_2_-specific string) is a substring of a string in *T* (condition 1) and it is not a substring of any string in *R* (condition 2). Moreover, strings in $S_{t_{1}}$ ($S_{t_{2}}$, respectively) are substring-free in the sense that each string is not a substring of another one in the same set. We have to prove that any *t*_1_-specific string *x* cannot be a substring of any *t*_2_-specific string *y*, and vice versa (substring-free property). We will prove this by contradiction. Let us assume that *x* is a substring of *y*. By definition *y* is not a substring of *R*, which implies that *x* is a substring of *R*: indeed *y* being substring-free, it holds that any substring of *y* is a substring of *R*. But *x* being a *t*_2_-specific string, we obtain a contradiction. At this point, the vice versa is trivial to prove. □

## 3 Preliminary concepts

The FMD-index (Li, 2012) is a data structure based on the FM-index (Ferragina and Manzini, 2000), which indexes a set of strings and their reverse complements at the same time, allowing to perform search operations on the index. Differently from the bidirectional Burrows–Wheeler Transform (BWT; Lam *et al.*, 2009), which builds two FM-indices, the FMD-index builds a single FM-index for both strands. The FM-index of the collection $\left\{r_{1},\ldots ,r_{n}\right\}$ of strings of sample *R* is essentially made of the BWT of *R*, which is itself a permutation *B* of the symbols of *R* obtained from the Generalized Suffix Array (GSA) *SA* of *R*. Indeed, recalling that SA[i] is equal to (*k*, *j*) if and only if the *k*-suffix of string *r_j_* is the *i*-th smallest element in the lexicographic ordered set of all suffixes of the strings in *R*, then $B[i]=r_{j}[|r_{j}|-k]$, if SA[i]=(k,j) and $k<|r_{j}|$, or B[i]= $otherwise.

Given a string *Q*, all suffixes that have *Q* as a prefix appear consecutively in GSA, where they induce an interval [b,e) which is called *Q-interval*. Note that the difference *e—b*, also called the width of the *Q*-interval is equal to the number of occurrences of *Q* as a substring of some string r∈R. The backward extension operation of an arbitrary character *σ* applied to the *Q*-interval of a string *Q* allows to determine the *σQ*-interval in the index. The FMD-index also allows to apply a forward extension operation of an arbitrary character *σ* to a *Q*-interval of a string *Q* to determine the Qσ-interval in the index. The implementation of both forward and backward operations in the FMD-index is realized by constructing an FM-index for the collection *R* concatenated with the reverse-complement of each string in *R*.

By adopting the same notations as in Li (2012), we keep a triple [i,j,l] (called *bi-interval*) that encodes for the *Q*-interval [i,i+l] and the $\bar{Q}$-interval [j,j+l], where $\bar{Q}$ is the reverse complement of string *Q*. Whenever *l* = 0 the *Q*-interval (respectively, $\bar{Q}$-interval) is empty and string *Q* (respectively, $\bar{Q}$) does not occur in *I_R_*. We will use notation t[b:e] to denote an interval on string *t*, i.e. t[b:e] is a substring of *t*, whereas $\left[i_{b},j_{b},l_{b}\right]$ to denote the corresponding t[b:e]-interval on the index *I_R_*.

## 4 Algorithm for sample-specific string detection

We present a novel algorithm (Algorithm 1, Ping-Pong search) to solve the SFS problem between a set of reference strings and a single target string t∈T. Our algorithm computes substring-free *t*-specific strings with respect to the reference sample *R* using the FMD-index of *R*, from now on denoted *I_R_*.

**Algorithm 1:** Computing *t*-specific strings from FMD-index *I_R_*

**1 Function** PingPongSearch(*t, I_R_*)

2 b←|t|−1

3 $\left[i,j,l]\leftarrow \mathrm{init}(I_{R},t[b]\right)$//*init* function initializes an FMD-Index bi-interval representing a single character

4 **while**b≥0**do**

**5 while** l≠0∧b>0 **do**//Step 1 - Backward extension

6 b←b−1

7 $\left[i,j,l]\leftarrow \mathrm{backwardExtension}(I_{R},[i,j,l],t[b]\right)$

8 **if**l≠0∧b=0**then return**

9 e←b

**10** $\left[i,j,l]\leftarrow \mathrm{init}(I_{R},t[e]\right)$

**11 while** l≠0 **do**//Step 2 - Forward extension

**12** $\left[i_{b},j_{b},l_{b}]\leftarrow [i,j,l\right]$

13 e←e+1

14 $\left[i,j,l]\leftarrow \mathrm{forwardExtension}(I_{R},[i,j,l],t[e]\right)$

15 *Output*t[b:e]

16 $\left[i,j,l]\leftarrow [i_{b},j_{b},l_{b}\right]$

The following main property that is a direct consequence of the substring-free property of specific strings is used to define the generic iteration step of Algorithm 1.

Lemma 1. *Let R be a collection of strings with FMD-index I_R_ and let t be a string that does not exist in R. Let x be the rightmost t-specific string currently found in t, where*$x=t[b_{x}:e_{x}]$*. It must then be the case that any other t-specific string must begin before b_x_. Assume such a specific string y exists and starts at b_y_, it must then be the case that y is the shortest prefix of*$t[b_{y}:e_{x}-1]$*that does not occur in the index.*

Proof. By definition, two specific strings cannot start at the same position as one cannot be a substring of the other. Thus given *x* the rightmost occurrence of a specific string in *t*, the second rightmost occurrence *y* of a specific string must start to the left of *b_x_*, i.e. given $y=t[b_{y}:e_{y}]$ it must be that *b_y_* < *b_x_*. By the substring-free property $t[b_{y}:e_{x}]$ will not occur in the index as it contains the substring *x*, which does not occur in the index. On the other hand, it must be that *e_y_* < *e_x_* otherwise *y* includes *x* as *b_y_* < *b_x_*, which is not possible by definition of *x* and *y* as substring-free specific strings. Thus *e_y_* < *e_x_* which implies that *y* is a prefix of $t[b_{y}:e_{x}-1]$. Now, *y* must be the shortest such prefix not in the index, otherwise, it includes another specific string contradicting the substring-free property, thus concluding the proof of the Lemma. □

Based on the previous Lemma, given the interval $\left[b_{x}:e_{x}\right]$ of the last detected specific string, the algorithm will start looking for a new occurrence of a specific-string from the end position $e_{x}-1$.

More precisely, the algorithm keeps track of two search positions *b* and *e* inside *t* which, respectively, represent the start and end of a substring of *t* that may or may not exist in *I_R_* and uses the constant-time forward and backward extension operations defined on the FMD-index (Li, 2012).

Given the index *I_R_* and a triple [i,j,l] encoding a *Q*-interval and $\bar{Q}$-interval, the algorithm alternates between extending the *Q*-interval backward (step 1, lines 5–7) and forward (step 2, lines from 11 to 14) to find *t*-specific strings. Figure 1b illustrates how the algorithm iterates over an input string *t*.

During each iteration of step 1, the algorithm backward extends the t[b:e]-interval of *I_R_* with t[b−1] until the backward extension in the index *I_R_* with t[b−1] is not possible. In other words, this is equivalent to finding the left maximal match ending at position *e* and extending it one base on the left. Now t[b−1:e] is a substring of *t* that is specific to *t*. However, such a substring is not necessarily the shortest, since one of its prefixes may also be specific.

Step 2 initializes *e* to *b—*1 and then keeps incrementing *e* by one position at a time, and performs a forward extension in *I_R_* for the prefix t[b−1:e] for each increment. If the forward extension with t[e+1] is not possible in *I_R_*, the algorithm stops and returns t[b−1:e+1] as the shortest string beginning from position *b—*1 that’s not in *I_R_*. In other words, we are looking for the longest right maximal match starting at position *b—*1 and then we are extending it one base to the right. We note that Algorithm 1 outputs substring t[b:e] since *b* (resp. *e*) has been already decremented (resp. incremented) previously in the corresponding while (i.e. step 1 for *b* and step 2 for *e*). Finally, since substring t[b−1:e] is not *t*-specific and is in the index, it could be extended to the left to compute a new *t*-specific which will eventually overlap the last computed *t*-specific t[b−1:e+1]. Line 16 initializes this process. Observe that Algorithm 1 may compute the same SFS multiple times when processing a string *t*; however, the output is still a set of *t*-specific strings without duplicates.

Theorem 1. *Algorithm 1 solves the SFS problem for a string t w.r.t. a reference set R in time*$\sum_{s\in S_{t}}O(|s|\times occ_{s}+|t|)$*, where occ_s_ is the number of times a string s is output by Algorithm 1 when processing t.*

Proof. We start by proving correctness and then time complexity. Based on Lemma 1, the Algorithm searches for a new specific string starting from the end position *e_x_* of the last detected specific string *x*. The correctness relies on the fact that Algorithm 1 visits from right to left each position *b* of the prefix of length *e_x_* of the input string *t* maintaining the following invariant property: the Algorithm outputs the shortest prefix t[b:e] of $t[b:e_{x}-1]$ which does not occur in the index *I_R_* (if such a string exists). Based on Lemma 1, this invariant property allows us to state that the Algorithm for any position *b* outputs the *t*-specific string starting at that position (which is unique by the substring-free property) if any; since all positions of the input string are processed by the algorithm, all possible specific strings are output in the end. We now show the invariant by analyzing a single iteration. Assume that *b* is a position such that t[b:e] is a *t*-specific string computed when the algorithm visits such a position of *t*. Now, let *k* be the smallest integer (with *k* < *b*) such that t[b−k:e−1] is the next string *x* not in the index. This is easily detected by backward extension, i.e. by iterating *k* times the loop from line 5 to 7 of the Algorithm. After finding *k*, the algorithm sets k′=0 and computes whether t[b−k:b−k+k′] is in the index for increasing values of k′ and stops as soon as t[b−k:b−k+k′] is not in the index thus computing the shortest prefix of t[b−k:e−1] not in the index. This concludes the proof of the invariant.

To prove Algorithm 1 time complexity, observe that it performs a number of backward extensions which is equal to the length of the string *t*, while it performs a number of forward extensions that is $O(l_{b})$ for *l_b_* being the length of the specific string retrieved from position *b* of *t*. Thus the time complexity easily follows from the above observation. □

### 4.1 Relaxed Ping-pong Search: a faster heuristic search algorithm

Observe that by Theorem 1 the worst-case time required to solve the SFS problem on a single string *t* is $O(n^{2})$ for *n* being the length of the string *t*, assuming that the index *I* is already available. Note that in the formula $\sum_{s\in S_{t}}O(|s|\times occ_{s}+|t|),\;|s|$ can be *O*(*n*) in the worst case and $\sum_{s\in S_{t}}O(|s|)$ can achieve the bound of $O(n^{2})$ since the strings in $S_{t}$ span positions of the string *t* that are overlapping and we can have *O*(*n*) strings in $S_{t}$ each of length *O*(*n*). See Supplementary Section S1.1 for an example. This clearly implies a quadratic time for solving the SFS problem when the input is no longer a single string *t* but a collection *T* of strings of total length *n*.

We consider a simple variant of Algorithm 1 that leads to a linear time complexity by avoiding the computation of specific strings that occur in overlapping positions of the original string *t*. The variation is simply obtained from the pseudo-code of Algorithm 1 by deleting instruction 12 and replacing line 16 with the instruction $\left[i,j,l]\leftarrow \mathrm{init}(I_{R},t[b-1]\right)$. This implies that the search procedure of *t*-specific strings starts from one position to the left of the beginning of the last detected string in *t*. We call this procedure the *relaxed Ping-pong Search*.

It is easy to verify that the relaxed version of Algorithm 1 is linear in the size of string *t*. Indeed, in the worst case, it performs two index queries per symbol of the input string: each character is searched in the index one time during the backward extension and one time during the forward extension (see Fig. 1b). Formally, when estimating the formula $\sum_{s\in S_{t}}O(|s|\times occ_{s}+|t|)$ of Theorem 1 in this variant, strings in $S_{t}$ occur in positions of *t* that are disjoint and thus in the worst case the sum of the sizes of strings in $S_{t}$ is $\sum_{s\in S_{t}}O(|s|\times occ_{s})=O(|t|)$, thus proving that the time complexity of the algorithm is linear in the size of the input string.

### 4.2 Relaxed output set upper-bounded by the edit distance

The edit-distance is a well-known measure in the comparison of two genome sequences. By counting the minimum number of nucleotide insertions, deletions and changes that transform a genome *t* into *r*, the edit distance between *t* and *r*, denoted by *D*(*t*, *r*) is clearly an upper bound for the number of positions with variations in *t* w.r.t. to *r*. In the following, we show that for a pair of strings *t* and *r*, each *t*-specific string returned by the relaxed version of Algorithm 1 corresponds to at least one edit operation that changes *t* into *r*, thus showing that *D*(*t*, *r*) is an upper bound on the size of its output set. Observe that the relaxed version of Algorithm 1 computes a subset of the *T*-specific strings w.r.t. *R* that has the substring-free property.

Theorem 2. *Given two strings t and r*, $\left|S_{t}\right|$*the size of the set of strings S_t_ returned by the relaxed Ping-pong search with respect to r, then*$\left|S_{t}|\leq D(t,r\right)$.

Proof. Since the set *S_t_* consists of string induced by nonoverlapping intervals of sequence *t*, any edit operation changes $\left|S_{t}\right|$ by at most 1. The minimum set of edit operations to convert *t* to *r* [i.e. *D*(*t*, *r*)] will transform *t* = *t*_0_ into successive strings $t_{1},t_{2},\ldots ,t_{D(t,r)}$ and eventually $t_{D(t,r)}=r$. For each operation, the successive sets of relaxed Ping-pong strings for $t_{2},\ldots$ change in cardinality by at most 1, i.e. $||S_{t_{i}}|-|S_{t_{i+1}}||\leq 1$ for 0≤i<D(t,r). Observe that D(t,r)=0 implies $|S_{t}|=0$ thus $|S_{t_{D(t,r)}}|=0$. Thus, total size of $\left|S_{t}\right|$ could not have been more than *D*(*t*, *r*) to start. □

### 4.3 Implementation details

**Fig. 2.**
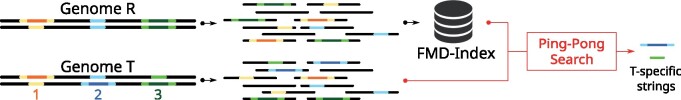
Illustration of sample-specific string detection. Two genomes *R* and *T* are depicted. With respect to genome *R*, site 1 has no variation in *T*, site 2 is a heterozygous insertion in *T*, and site 3 is a heterozygous deletion in *T*. Our pipeline aims to detect *T*-specific strings by (**a**) indexing the reads sequenced from *R* with an FMD-Index and (**b**) analyzing the reads sequenced from *T* with our novel *Ping-pong search* algorithm. We note that, for ease of presentation, we depict at the end of the pipeline a single *T*-specific string per site even though multiple *T*-specific strings may actually be reported for each site

We implemented Algorithm 1 in C++ based on code from ropeBWT2 (Li, 2014). After creating the index of the reference set *R*, our code executes Algorithm 1 on each target string t∈T while also keeping track of the number of times each specific string is seen (Fig. 2). As each target, string can be processed independently, our code is embarrassingly parallel. Once all target strings have been analyzed, a postprocessing step combines the smaller solutions into the final solution of the SFS problem. In order to remove specific strings produced by sequencing errors when our method is run on WGS data, the postprocessing step can filter out all the specific strings occurring less than *τ* times, with *τ* being a user-defined cutoff. Our implementation is freely available at https://github.com/Parsoa/PingPong.

## 5 Results

### 5.1 Specific string detection in simulated human HiFi trio

We used simulations to test the performance of our proposed method in detecting *de novo* SVs in WGS trios (i.e. proband, mother and child). We mutated the GRCh38 genome randomly with 6115 insertions and deletions from the 1KG project (Chaisson *et al.*, 2019) to produce two haplotypes for each parent. We limited the simulations to chromosomes 1–5. We then simulated the child genome by inheriting variants from the parents and considering recombination inside each chromosome. Finally, we introduced an additional 17 595 randomly generated *de novo* structural variants equally divided between insertions, deletions and inversions into the child genome impacting 7 913 593 base-pairs. See Supplementary Figure S1 for the distribution of lengths of simulated SVs.

We simulated reads from the father, mother and child genomes at different coverage levels (5×, 10×, 20× and 30×) for each haplotype using PBSIM (Ono *et al.*, 2013) with sequencing error rate and read length distribution similar to real HiFi data. Specifically, we sampled these parameters from the HGSVC2 PacBio HiFi reads for the HG00733 sample (Porubsky *et al.*, 2020) with the error rate averaging at 0.1%. All three samples were error corrected using ntEdit (Warren *et al.*, 2019) to remove sequencing errors. The combined reads of the father and mother were indexed using FMD-index and we searched for child-specific strings using Algorithm 1 (exact version).

**Fig. 3.**
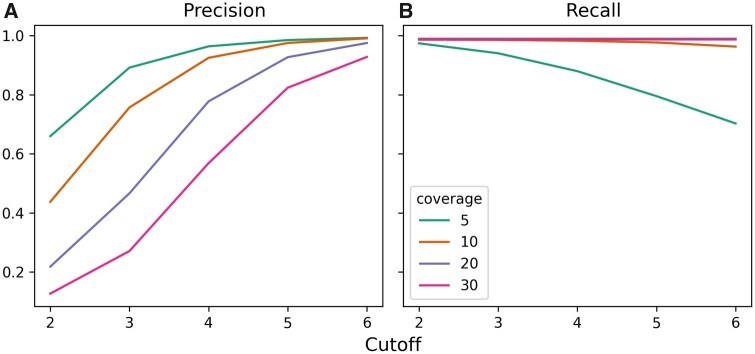
Precision (**a**) and recall (**b**) calculated for different coverage levels (5×, 10×, 20× and 30× per haplotype) and cutoff values 2≤τ≤6 in simulation

We measured the accuracy of the method using two metrics of *recall* and *precision*. Recall is defined as the percentage of *de novo* variants that are covered with child-specific strings and precision is defined as the percentage of child-specific strings that cover a *de novo* variant. We test the performance of the method for different *τ* cutoff values (2≤τ≤6) to study the relationship between this parameter and sequencing coverage levels and to measure our method’s sensitivity (Fig. 3). While the high coverage simulations (30×, 20× and 10×) have constantly high recall rates regardless of *τ*, the low-coverage 5× sample’s recall drops significantly with larger cutoff values.

We analyzed the child-specific strings from the 30× simulation using *τ* = 5 in more detail. A total of 14 381 350 child-specific strings were retrieved with 2 052 144 remaining after filtering low-abundance strings. The selected child-specific strings achieved >98% recall and 82% precision at recovering simulated *de novo* SVs. To better demonstrate the usefulness of child-specific strings, we compared the performance of the strings generated using both the exact and relaxed versions of Algorithm 1 on the 30× simulation against child-specific *k*-mers of fixed sizes 32 and 101 bp with abundance of at least 5. Child-specific *k*-mers were calculated using KMC3 (Kokot *et al.*, 2017) by subtracting the set of parent *k*-mers from the set of child *k*-mers. We calculated precision and recall by mapping the *k*-mers and SFS strings to the child haplotypes with BBMap (Bushnell, 2014). We observe that SFS consistently performs better than fixed-length *k*-mers. The results can be seen in Table 1.

**Table 1.** Comparison of performance of SFS and fixed-length *k*-mers in the 30× simulation with *τ* = 5

	Sequences			Variants	
Method	Total	Covering	Precision (%)	Covered	Recall (%)
Alg. 1 (exact)	1 690 675	2 052 144	82.38	17 367	**98.70**
Alg. 1 (relax)	377 007	366 390	**97.18**	17 365	98.69
32-mers	5 089 147	3 053 969	59.91%	17 317	98.42
101-mers	7 060 167	3 453 211	48.91%	17 348	98.59

The bold values represent the best performance in each column.

We further analyzed the qualities of the alignments of child-specific strings against all three genomes in the trio (Supplementary Fig. S2). Alignment quality is evaluated based on the number of bases that do not match. More than 83% of child-specific strings map perfectly to the child genome, and no (zero) string has a mismatch-free mapping to either parent genomes, indicating that the strings are truly child-specific.

Finally, we re-ran the simulation at 30× coverage without incorporating any sequencing errors in the trio. In this scenario, the simulated SVs are the sole source of novel sequences in the child compared to the parents and therefore we expect every recovered SFS to cover a variant. Analyzing the 1 720 395 child-specific strings retrieved in this scenario indeed yields a precision of 100.0%. However, the recall remains the same as in the case with sequencing errors, at 98.70%. This is because some variants do not produce novel sequences and thus cannot be captured with our approach.

### 5.2 Specific string detection in real human HiFi data

We performed an extensive evaluation of sample-specific strings using real HiFi data to assess their ability to compare two individuals of different populations. We considered the HG00733 child (Puerto Rican trio) and the NA19240 child (Yoruba trio). For both these individuals, the HGSVC2 (Porubsky *et al.*, 2020) provides a PacBio HiFi 30× sample. Supplementary Figure S3 reports the length distribution of the considered samples.

After correcting both samples with ntEdit (Warren *et al.*, 2019), we indexed the NA19240 sample and we searched for HG00733-specific strings (from now on we will refer to these strings simply as ‘specific’) using both the exact and the relaxed version of our algorithm. Supplementary Table S1 reports the running times and the peak memory usage of our pipeline; the creation of the FMD-Index was the most time-consuming step. Based on the results on simulated data (Fig. 3) and the coverage of the two samples (30×), we considered all specific strings occurring more than five times. The main goal of this postfiltering is to remove from downstream analyses specific strings that with high probability are the result of sequencing errors. Using the exact (relaxed, respectively) version of our algorithm, we retrieved 34 219 149 (7 125 436, respectively) strings. Supplementary Figure S4 reports information on the lengths and the abundances of these strings. As expected, the exact version of our algorithm is slower and retrieves more strings than the relaxed one.

### 5.2.1 Contigs-based analysis

**Fig. 4.**
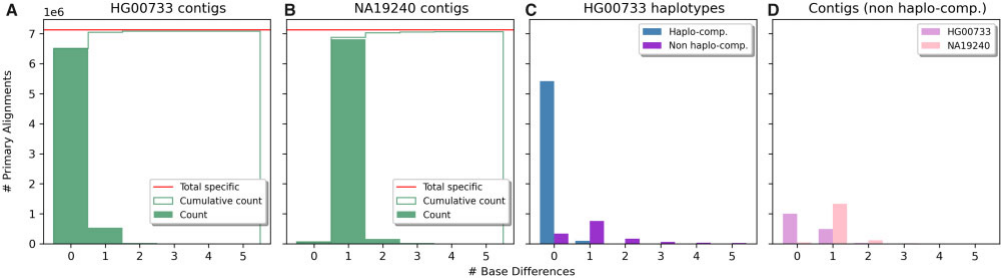
Results on exact HG00733-specific strings. (**a, b**) Comparison of the quality of specific strings alignments computed against the HG00733 contigs (**a**) and the NA19240 contigs (**b**). (**c**) Comparison of the qualities of the specific string alignments representing (Haplo-compatible) and not representing (Nonhaplo-compatible) a specific portion of HG00733 haplotypes. (**d**) Comparison of the qualities of nonhaplo-compatible specific string alignments computed against the HG00733 contigs and the NA19240 contigs. Quality is expressed as number of base differences (mismatches, insertions, deletions and clips)

We first analyzed the quality of HG00733-specific strings by checking whether they are effectively specific to the HG00733 child. To do so, we aligned the strings to the contigs provided by the HGSVC2 consortium of the two individuals and we counted base differences (substitutions, insertions, deletions and clips) within alignments. We mapped strings shorter than 500 bp with BBMap (Bushnell, 2014) and longer ones with minimap2 (Bushnell, 2014). We used two different aligners since BBMap showed higher sensitivity in mapping short (<50 bp) strings. Figure 4a and b shows the results of this analysis for the exact version of our algorithm (see Supplementary Fig. S5 for the relaxed results). A total of 33 964 009 specific strings were mapped to the HG00733 contigs and 27 326 747 (80%) of these were aligned perfectly, i.e. without any base difference. On the other hand, 33 932 307 specific strings were mapped to the NA19240 contigs but only 158 094 (0.4%) of these were aligned perfectly.

To summarize the results of this contigs-based analysis, we introduced the *C-precision* (contigs-based precision) metric. Based on the alignments to the contigs, it computes the fraction of HG00733-specific strings that align perfectly to HG00733 contigs and not perfectly to NA19240 contigs. Out of 27 326 747 specific strings aligned perfectly to HG00733 contigs, 132 031 aligned perfectly also to NA19240 contigs. The exact version of our algorithm therefore achieved a C-precision of 79.47%. On the other hand, the relaxed version achieved a C-precision of 90.61%. This was expected since the relaxed version of our algorithm retrieves a lower number of specific strings easily achieving a higher precision at the expense of, as we will see in the next section, a lower recall (see Table 2). This analysis shows that the strings output by our algorithm are effectively specific to the HG00733 and may be effectively used to characterize differences between the two individuals.

**Table 2.** Variant analysis on real human HiFi data

Metric		Method	Missed	Total	Hits (%)
Recall	SNPs	Alg. 1 (exact)	39 354	3 147 410	**98.75**
		Alg. 1 (relaxed)	112 940		96.41
		31-mers	243 363		92.27
		101-mers	46 143		98.53
	indels	Alg. 1 (exact)	31 426	716 226	**95.61**
		Alg. 1 (relaxed)	120 313		83.20
		31-mers	131 591		81.63
		101-mers	32 944		95.40
	SVs	Alg. 1 (exact)	1 521	20 775	**92.68**
		Alg. 1 (relaxed)	2 948		85.81
		31-mers	4 912		76.36
		101-mers	1 978		90.48
	All	Alg. 1 (exact)	72 301	3 884 411	**98.14**
		Alg. 1 (relaxed)	236 201		93.92
		31-mers	379 866		90.22
		101-mers	81 065		97.91
H-precision		Alg. 1 (exact)	9 093 407	34 219 149	73.43
		Alg. 1 (relaxed)	1 583 684	7 125 436	**77.77**
		31-mers	28 561 768	97 975 734	70.85
		101-mers	120 109 600	387 221 925	68.98
C-precision		Alg. 1 (exact)	7 024 433	34 219 149	79.47
		Alg. 1 (relaxed)	669 324	7 125 436	**90.61**
		31-mers	23 170 031	97 975 734	76.35
		101-mers	84 211 940	387 221 925	78.25

*Note*: Recall is the fraction of known alleles specific to HG00733 (w.r.t. NA19240) overlapped by at least one HG00733-specific string (or specific *k*-mer). For the sake of completeness, we reported the recall values for alleles coming from SNPs, indels (2–49 bp), and SVs (≥50 bp), as well as all considered specific alleles. H-precision (Haplotype-aware precision) is the fraction of HG00733-specific strings (or HG00733-specific *k*-mers) representing a portion of its haplotypes that is specific w.r.t. the NA19240 haplotypes. C-precision (Contig-based precision) is the fraction of HG00733-specific strings (or *k*-mers) aligning perfectly only to HG00733 contigs (and with errors to NA19240 contigs).

The bold values represent the best performance in each column.

### 5.2.2 Haplotypes-based analysis

We evaluated the effectiveness of HG00733-specific strings in covering variant alleles that are specific to the considered individual. To do so, we considered the phased callset provided by the HGSVC2 consortium (Ebert *et al.*, 2020) and, after filtering out overlapping variations, we extracted for each variation and for each haplotype the set of alleles that are present in the HG00733 child but not in the NA19240 (we will refer to these alleles as *HG00733-specific* or simply *specific alleles*). Therefore, each variation may have 0, 1 or 2 specific alleles. For instance, if a variation has genotype 0|2 in the HG00733 child and 1|1 in the other child, we considered alleles 0 and 2 as specific to the HG00733. Table 2 (column *Total*) reports the number of specific alleles we considered in our analysis. We classified each allele with respect to the type of its originating variant [following the classification in Ebert *et al.* (2020)]: SNPs, indels (insertions and deletions of 1–49 bp), and SVs (insertions and deletions of ≥50 bp), which include copy number variants and balanced inversion polymorphisms.

Considering the entire set of known variations, we built the haplotypes of the HG00733 individual using BCFtools and then we aligned the HG00733-specific strings (occurring more than five times) to them using BBMap (strings ≤500 bp) and minimap2 (strings > 500 bp). Finally, we used BEDtools (Quinlan and Hall, 2010) (*intersect* sub-command) to find the overlaps between the alignments and the considered alleles.

We evaluated the quality of our specific strings in terms of *recall*, i.e. number of specific alleles effectively intersected by at least one alignment, and *H-precision* (Haplotype-compatible precision), i.e. the number of specific strings representing a specific portion of a haplotype of the HG00733 child. By ‘specific portion’, we mean a subsequence of an HG00733 haplotype induced by a set of variations that is different from the subsequence of any NA19240 haplotype induced by the same set. Table 2 reports the results of this analysis. We introduced the H-precision measure since close alleles (especially SNPs) on a haplotype of one individual may result in a specific string even when neither alleles are specific. Indeed, a set of close alleles may be shared between two individuals but in one individual they may be on the same haplotype whereas in the other one on different haplotypes. Consider for instance two nearby variants with genotypes 0|1 and 0|1 in one individual and 1|0 and 0|0 in the other. In this case, the haplotype containing alleles 1 and 1 is specific to the first individual even though single alleles are not.

Remarkably, the set of specific strings computed by our method (exact version) intersect most of the HG00733-specific alleles (>98%), covering nearly all alleles coming from SNPs and indels (>98% and >95%, respectively) and most of alleles coming from SVs (>92%). We observed that a majority of the variants not covered by the sample-specific strings were indels in stretches of A or T sequences, likely addressable through improvements in homopolymer error correction.

Out of the 34 219 149 specific strings retrieved by the exact version, 73.43% of them represent a specific portion of the HG00733 haplotypes (H-precision). Figure 4c reports the comparison in terms of base differences between the alignments representing specific portions of the haplotypes (denoted as ‘haplo-compatible’) and those that do not (denoted as ‘non haplo-compatible’). As expected, the vast majority of the haplo-compatible strings are aligned perfectly to the haplotypes whereas the vast majority of nonhaplo-compatible strings are aligned with errors.

To better investigate why ∼27% of the specific strings align well to the HG00733 haplotypes but do not represent a specific portion of them (accordingly to the considered VCF), we aligned those strings to the contigs of the two individuals. Figure 4d reports the results of this analysis. 2 885 356 strings were aligned perfectly to the HG00733 contigs whereas only 208 330 were mapped perfectly to the NA19240. Moreover, ∼1.8 million specific strings align perfectly to the HG00733 contigs but not to its haplotypes. This leads us to conjecture that a portion of those strings corresponds to true variants missing from the VCF.

Results on strings retrieved by the relaxed algorithm follow the same trend (see Supplementary Fig. S5). They, however, achieve higher H-precision and lower recall than the exact version (see Table 2), likely due to a lower number of strings returned. Moreover, the relaxed algorithm may fail in covering close variations: if two variations are too close to each other, strings retrieved by our relaxed algorithm may cover only the right-most variation (due to its right-to-left traverse of input strings). See Supplementary Figure S6 for an example.

To put our results in perspective, we compared them with a *k*-mer method. Similarly to an HG00733-specific string, an *HG00733-specific k-mer* is a *k*-mer occurring in the HG00733 sample and not in the NA19240. To compute the set of specific *k*-mers, we first counted all *k*-mers occurring more than five times in the two samples independently with KMC3 (Kokot *et al.*, 2017) and then we retrieved the *k*-mers present only in the HG00733 sample by subtracting the two sets (kmc_tools *kmers_subtract* operation). A total of 97 975 734 HG00733-specific *k*-mers (*k* = 31) were retrieved. We then mapped those to HG00733 haplotypes with BBMap and evaluated their recall and H-precision similarly to HG00733-specific strings. Table 2 reports the results of this analysis. HG00733-specific 31-mers achieved lower recall and H-precision than HG00733-specific strings, although their computation is faster (8 h for *k*-mers versus 28–37 h for Ping-Pong, see Supplementary Table S1). The poor performance of 31-mers can be explained by their length: a 31-mer located at a variant position might occur elsewhere in the genome, whereas a longer string would be unique. We note that long (>500 bp) HG00733-specific strings retrieved by the exact algorithm cover ∼1.5% of indels and SVs not covered by shorter ones, proving that longer strings are sometimes needed to effectively cover a variation.

For this reason, we also performed an analysis using longer *k*-mers (*k* = 101). A total of 387 221 925 101-mers were retrieved. However, BBMap failed to align that many *k*-mers in reasonable time. We therefore aligned them with BWA-MEM (Li, 2013) and computed their recall and H-precision. Results of this analysis can be found in Table 2. Thanks to their length, 101-mers are able to cover more variations than 31-mers but not as many as our (exact) specific strings which are of variable length, sometimes longer than 101 bp. For instance, Supplementary Figures S7 and S8 show two examples of variants covered by specific strings and not by specific 101-mers, highlighting the biological usefulness of our method. Moreover, 101-mers are less precise than (exact) HG00733-specific strings: indeed, due to their overlapping nature, a false variant (e.g. a sequencing error) will in the worst case yield 101 false specific 101-mers. We therefore mapped the specific *k*-mers to the contigs of the two individuals and we computed their C-precision (fraction of specific *k*-mers mapping perfectly only to HG00733 contigs). Similarly to specific strings, C-precision of 31-mers and 101-mers is higher than their H-precision (see Table 2), proving one more time that the considered VCF may be incomplete.

Finally, in an attempt to reduce the number of strings obtained using the *k*-mer method, we assembled the 31-mers and the 101-mers into unitigs [which correspond to maximally extending *k*-mers using their (k−1)-overlaps and stopping at any variation] using BCALM2 (Chikhi *et al.*, 2016) and we computed their recall and H-precision. Results of this analysis can be found in Supplementary Table S2. Surprisingly, assembling the *k*-mers into unitigs did not improve their overall accuracy.

## 6 Discussion

We have here presented a novel algorithm called Ping-Pong search for finding SFS strings with the primary objective of performing comparative genome analysis between two groups of whole-genome sequenced samples. We have shown that these SFS strings capture a comprehensive representation of genomic variation between samples of interest. In practice, the proposed approach is capable of finding sequences that span the breakpoints of most variants specific to each sample.

The proposed approach improves upon using fixed-length sequences (i.e. *k*-mers) for comparative genome analysis in three aspects: (i) higher recall: SFS sequences cover a higher fraction of true difference between two genomes than fixed-length *k*-mers. This is mainly due to their variable-length nature which increases our power in finding strings representative of differences between genomes in repetitive regions (e.g. segmental duplications). (ii) higher precision: our experiments have indicated that SFS sequences have a higher precision than fixed-length *k*-mers (*k* = 31 or 101). (iii) specificity: our exact algorithm returned between 3× and 10× less strings than *k*-mers, making results more amenable to further analysis. As a motivating example, we could not exhaustively map the results of the 101-mer analysis in reasonable time (<1 week).

Our method also has several major advantages over traditional mapping-based approaches for comparative genome analysis. First, it is not dependent on a prior knowledge of variants in each sample and thus, its performance is not impacted by the biases in variant prediction methods. Second, the proposed approach does not require mappings of the reads, hence, ambiguities in read mappings or biases in mapping algorithms will not impact the results of the proposed method.

One of the main limitations of the proposed method is its reliance on reads with low sequencing error (e.g. HiFi reads). To be able to accurately predict SFS strings from reads with sequencing errors, we need to utilize an error correction tool such as ntEdit. This method is not expected to translate well to higher error-rate long reads, unless correction yields nearly perfect reads. Another downside is the 3× longer running time of the relaxed algorithm compared to *k*-mers. This longer runtime is mainly due to the overhead of building the FMD index. We note that the FMD index can be replaced by a more efficient implementation that offers the same backwards and forward extension operations, if such a data structure or implementation becomes available, thus improving the performance of the method.

We believe there are many applications and possible future research directions for SFS. An obvious application of the experiments presented in this manuscript would be the discovery of *de novo* variants in the child-sample in genomic trios (Section 5.1). Another potential application would be the discovery of somatic variants between whole-genome sequences of tumor and normal tissues. Furthermore, as SFS strings will capture any variant as long as it produces a genomic sequence not present in the FMD index, our method could be used as an orthogonal approach to catalog all variants in a given sample against the reference genome. Note that, this mapping-free variant calling approach against reference genome would be significantly faster than the comparative analysis scenario as only the reference genome needs to be FMD-indexed. Another application of the proposed approach is the discovery of variants between two samples that are missed using traditional mapping-based approaches. The SFS remaining after filtering the strings covering the predicted variants using mapping-based approaches may indicate potential novel variants missed by traditional mapping-based approaches.

Furthermore, a potential venue for more theoretical research could be to investigate the connection between SFS strings and other related but different concepts in stringology, such as maximal exact/unique matches, minimum unique substrings (Ye *et al.*, 2010) and shortest uncommon superstrings.

## Funding

This project has received funding from the European Union’s Horizon 2020 research and innovation program under the Marie Skłodowska-Curie grants agreements numbers [872539] and [956229], ANR Inception (ANR-16-CONV-0005) and ANR Prairie (ANR-19-P3IA-0001). This work has also been supported in part by NSF award DBI-2042518 to F.H.

*Conflict of Interest*: The authors declare no conflicts of interest.

## Supplementary Material

vbab005_Supplementary_Data
